# Reduction, Prevention, and Control of *Salmonella enterica* Viable but Non-culturable Cells in Flour Food

**DOI:** 10.3389/fmicb.2020.01859

**Published:** 2020-08-21

**Authors:** Yanmei Li, Tengyi Huang, Caiying Bai, Jie Fu, Ling Chen, Yi Liang, Kan Wang, Jun Liu, Xiangjun Gong, Junyan Liu

**Affiliations:** ^1^Department of Haematology, Guangzhou Women and Children’s Medical Center, Guangzhou Medical University, Guangzhou, China; ^2^Department of Laboratory Medicine, The Second Affiliated Hospital of Shantou University Medical College, Shantou, China; ^3^Guangdong Women and Children Hospital, Guangzhou, China; ^4^Guangdong Province Key Laboratory for Green Processing of Natural Products and Product Safety, School of Food Science and Engineering, South China University of Technology, Guangzhou, China; ^5^Overseas Expertise Introduction Center for Discipline Innovation of Food Nutrition and Human Health (111 Center), Guangzhou, China; ^6^Guangdong Zhongqing Font Biochemical Science and Technology Co., Ltd., Maoming, China; ^7^Research Center of Translational Medicine, The Second Affiliated Hospital of Shantou University Medical College, Shantou, China; ^8^School of Materials Science and Engineering, South China University of Technology, Guangzhou, China; ^9^Department of Civil and Environmental Engineering, University of Maryland, College Park, MD, United States

**Keywords:** *Salmonella enterica*, propidium monoazide, viable but non-culturable, environmental stress conditions, food system

## Abstract

The processing and storage conditions of flour food inevitably pose environmental stress, which promote bacteria to enter a viable but non-culturable (VBNC) state. The existence of VBNC cells causes false-negative detection in traditional culture-based detection methods, resulting in food quality and safety issues. This study aimed at investigating the influence factors including nutrition, acid, salt, and temperature for the entry into a VBNC state of *Salmonella enterica* and an efficient detection method. During induction with multi-stress conditions, nutrition starvation antagonizes with low-level acidity. Besides, high-level acidity was considered as an inhibitor for VBNC induction. Four inducers including nutrition starvation, salt stress, low-level acidity, and low temperature were concluded for a VBNC state. In addition, the keynote conditions for *S. enterica* entering a VBNC state included (i) nutrient-rich acidic environment, (ii) oligotrophic low-acidity environment, and (iii) oligotrophic refrigerated environment. Based on the keynote conditions, the environmental conditions of high acidity (1.0% v/v acetate) with low temperature (−20°C) could successfully eliminate the formation of *S. enterica* VBNC cells in flour food. In addition, combining with propidium monoazide pretreatment, PCR technology was applied to detect *S. enterica* VBNC cells. The sensitivity of the PMA–PCR technology was 10^5^ CFU/ml in an artificially simulated food system. The results derived from this study might aid in the detection and control of VBNC state *S. enterica* in flour food products.

## Highlights

- PMA-PCR technology was applied to detect *Salmonella enterica* viable but non-culturable (VBNC) cells with a sensitivity of 10^5^ CFU/ml.
- The keynote conditions for *S. enterica* entering a VBNC state include nutrient-rich acidic environment, oligotrophic low-acidity environment, and oligotrophic refrigerated environment.
- A high acidity (1.0% v/v acetate) plus frozen temperature (−20°C) environmental condition could successfully inhibit the formation of *S. enterica* VBNC cells and eliminate it in flour food.

## Introduction

In the food industry, flour food is frequently contaminated by foodborne bacteria including *Staphylococcus aureus*, *Salmonella enterica*, and *Escherichia coli O157* (Kirk et al., 2015; Lin et al., 2017; Miao et al., 2017c; Zhao et al., 2018a,b; Liu et al., 2019; Sharma et al., 2019). Foodborne *S. enterica* is a typical zoonotic pathogen with multiple toxic effects including invasiveness, endotoxin, and enterotoxin (Eng et al., 2015; Bao et al., 2017b,c; Xie et al., 2017a; Jia et al., 2018; Wen et al., 2020). Recently, studies had reported that *S. enterica* can form viable but non-culturable (VBNC) cells under certain environmental stresses (e.g., low temperature, salt stress, nutrient starvation) (Roszak et al., 1984; Chmielewski and Frank, 1995; Gupte et al., 2003; Kusumoto et al., 2012; Zeng et al., 2013; Morishige et al., 2017; Highmore et al., 2018). In addition to the natural environment, the generation of an *S. enterica* VBNC state also occurred during chlorination of wastewater or food (Oliver et al., 2005; Highmore et al., 2018). In the food industrial environment, the common non-ionic detergents and sanitizers were found to induce an *S. enterica* VBNC state formation (Morishige et al., 2013; Purevdorj-Gage et al., 2018; Robben et al., 2018). Besides, oxidation stress induced by non-thermal sterilization technologies had been confirmed to have a positive relationship with generation of VBNC *S. typhimurium* cells (Liao et al., 2018). Moreover, storage and complex components of food inevitably cause multistress conditions including high acidity and salt, nutrient starvation, and low temperature (Xu et al., 2011b; Liu et al., 2018a,b). Therefore, *S. enterica* VBNC cells could exist in the food industry and processing plants, and even food production. As the detection of foodborne pathogens in foods was based on colony-counting method, bacterial VBNC cells cause false-negative results and remain in food (Bao et al., 2017a; Xie et al., 2017b; Xu et al., 2018). Although VBNC cells have low activity, some frozen food has a long shelf life so that VBNC cells have enough time to metabolize causing food spoilage, which poses a certain safety hazard to human health (Fakruddin et al., 2013; Zeng et al., 2013; Highmore et al., 2018).

Although *S. enterica* VBNC cells could be resuscitated by favorable conditions, the recovery of VBNC cells from different environmental stresses requires different methods, such as temperature upshift (Gupte et al., 2003; Zeng et al., 2013), catalase ([Bibr B62][Bibr B33]), Tween 80 (Zeng et al., 2013), and nutrients (Roszak et al., 1984; Morishige et al., 2013). Therefore, the detection of *S. enterica* VBNC cells by resuscitation is infeasible. In recent years, combining propidium monoazide (PMA) treatment with nucleic acid amplification technologies has shown to be capable of rapidly detecting VBNC bacteria (Wang et al., 2011; Xu et al., 2011a,b, 2012a,b, 2016a,b; You et al., 2012; Liu et al., 2015, 2017; Jiang et al., 2016; Lin et al., 2016; Miao et al., 2016, 2017a,b, 2018; Ma et al., 2017; Wang et al., 2019).

In this study, the induction and control of VBNC state formation focused on specific environmental conditions were investigated (Zhang et al., 2013; Miao et al., 2017c; Xu et al., 2011a,c). Also, we applied the PMA–PCR method to detect the targeted gene *invA* of *S. enterica* VBNC cells in a food system (Xu et al., 2007, 2008a,b, 2009, 2010).

## Materials and Methods

### Bacteria Strains and DNA Extraction

The bacterial strains (Table 1) were grown in tryptic soy broth (TSB, Huankai Microbial, China) cultures at 37°C at 200 rpm for 24 h until further use. Then 1.5–2 ml of culture was used in DNA extraction by a DNA extraction kit (Dongsheng Biotech, Guangzhou) following the manufacturer’s instructions. Nano Drop 2000 (Thermo Fisher Scientific Inc., Waltham, MA, United States) was applied to measure the concentration of the extracted DNA for controlling the ratio value of OD_260_/OD_280_ from 1.8 to 2.0. All of the DNA samples were stored at −20°C until further use.

**TABLE 1:** Bacteria used in the study.

**Species**	**Source**
*Escherichia coli* O157:H7 ATCC47853	ATCC
*Escherichia coli* O157:H7 ATCC25922	ATCC
*Salmonella enterica* ATCC14028	ATCC
*Salmonella enterica* ATCC29629	ATCC
*Pseudomonas aeruginosa* ATCC27853	ATCC
*Staphylococcus aureus* 10071	Laboratory strain
*Listeria monocytogenes* ATCC19115	ATCC
*L. monocytogenes* ATCC19116	ATCC
*Lactobacillus casei*	Laboratory strain

### Induction of *S. enterica* VBNC Status

According to the food environmental condition, a total of three factors were selected as a single variable including nutrient, salt, and acid (Table 3). The designed orthogonal array was divided into 16 groups (Table 4), and the trend on the number of cultivable bacteria is used as an index to investigate the effect of external environmental pressure on the formation of *S. enterica* VBNC. The *S. enterica* VBNC status was induced by the 16 groups of conditions at low temperature (4° or −20°C). The overnight bacterial culture (∼10^8^ CFU/ml) was washed three times and resuspended by sterile saline. Besides, aliquots of these bacterial suspensions were separated into 1.5 ml tubes (∼20) to avoid the effect of repeated freeze–thaw. The viability of bacterial cells was characterized by the colony counting method, and the VBNC cells were determined by LIVE/DEAD BacLight^®^ kit (Thermo Fisher Scientific, United States) with fluorescence microscope after the culturable colonies no longer form on an agar medium. The culturable and viable cell enumerations were preformed every 3 days.

### PMA-PCR

The *S. enterica*-specific gene *invA* was selected as the target gene, and corresponding primers were designed (Table 2). The selected conserved regions were determined to be highly specific by sequence comparison on the Blast website. Primer Premier 5 was used to design primers for the PCR amplification reaction. All primers were synthesized by Guangzhou Aiji Biotechnology Co., Ltd. The mentioned bacterial DNA extraction was employed as a template for PCR amplification. The PCR assay was performed in a 25 μl volume with 1.6 μl of detection primer (50 μM) and 0.8 μl of accelerated primer (50 μM). The thermal profile for PCR was 94°C for 5 min, followed by the condition for 30 cycles: denaturation of 94°C for 30 s, primer annealing at 55°C for 30 s, and extension at 72°C for 1 min and a final extension cycle at 72°C for 7 min. A negative control was performed using sterile water instead of culture or DNA template. Finally, the specificity and sensitivity of the designed primer was determined by electrophoresis.

**TABLE 2:** Primer sequences of the target genes.

**Primer**	**Sequence (5′-3′)**	**Length**
*invA-*Ft	CACAAAGATGATAATGATGCCAATACTGGAAAGGGAAAGCC	41
*invA-*Bt	CCGTAGTAATAGTAGAAACACGACAGAGCGGAGGATAAA	39
*invA-*IF	TCATCGCACCGTCAAA	16
*invA-*IB	TGGCGGTATTTCGGTGGG	18

**TABLE 3:** External environmental factors during induction of the VBNC state of *Salmonella.*

**TSB (%)**	**NaCl (%)**	**Acetate (%)**
0	0.9	0
25	10	0.3
50	20	0.7
100	30	1

### PMA-PCR Detection on *S. enterica* VBNC Cells in a Food System

Twenty-five grams of crystal cake powder was added to 225 ml of sterile saline and autoclaved. The 1-ml overnight culture (∼10^8^ CFU/ml) was diluted 10 fold by adding 9 ml of sterile flour solutions to prepare the artificially contaminated food samples with different concentrations (10^7^–10). Before the extraction of bacterial DNA, artificially contaminated samples were pretreated according to the following steps: (1) 1 ml of the flour solution was centrifuged for 10 min at 1,000 rpm to remove the macroparticles, then the supernatant was centrifuged again at 12,000 rpm for 10 min to collect precipitations. (2) The precipitations were resuspended in 500 μl of sterile saline and then mixed with 125 μl of ethyl acetate for 2 min to remove impurities such as oil and fat. After centrifugation at 12,000 rpm for 10 min, the precipitations were washed once with 500 μl of TE buffer and twice with 500 μl of sterile saline.

The range of *S. enterica* VBNC cell concentration was adjusted from 10 to 10^6^ cells/ml. Subsequently, the propidium monoazide (PMA) dye was added to the flour samples until its concentration reached 5 μg/ml. After incubation at room temperature for 10 min in the dark, the samples were exposed to a 650 W halogen lamp with a distance of 15 cm for 5 min, which inactivated unbinding PMA molecules rather than PMA–DNA molecules. All the dyeing process was performed in an ice bath to prevent DNA damage. Subsequently, the DNA extraction and PCR detection of PMA-treated cells were performed.

## Results

### Induction of *S. enterica* VBNC State

**FIGURE 1:**
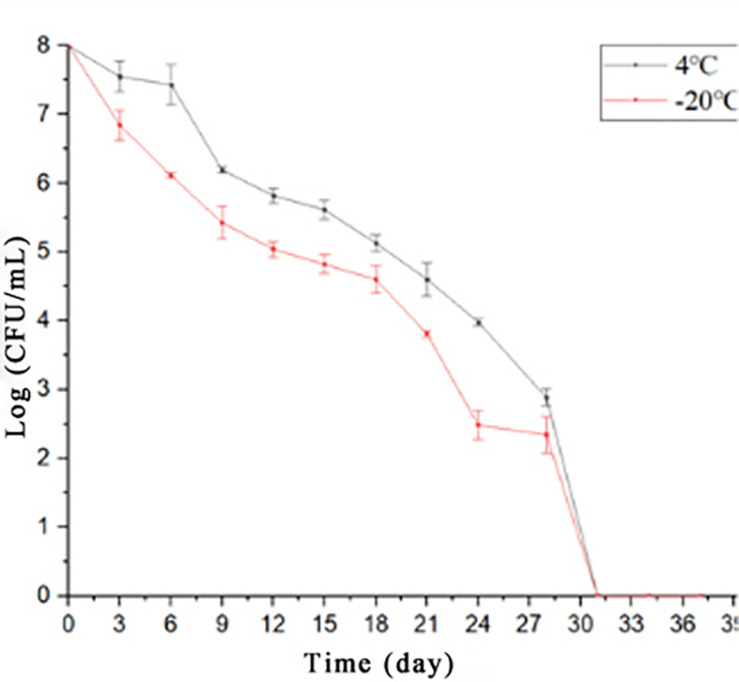
The culturable cells of foodborne *Salmonella* under low nutrients at 4° or −20°C.

**FIGURE 2:**
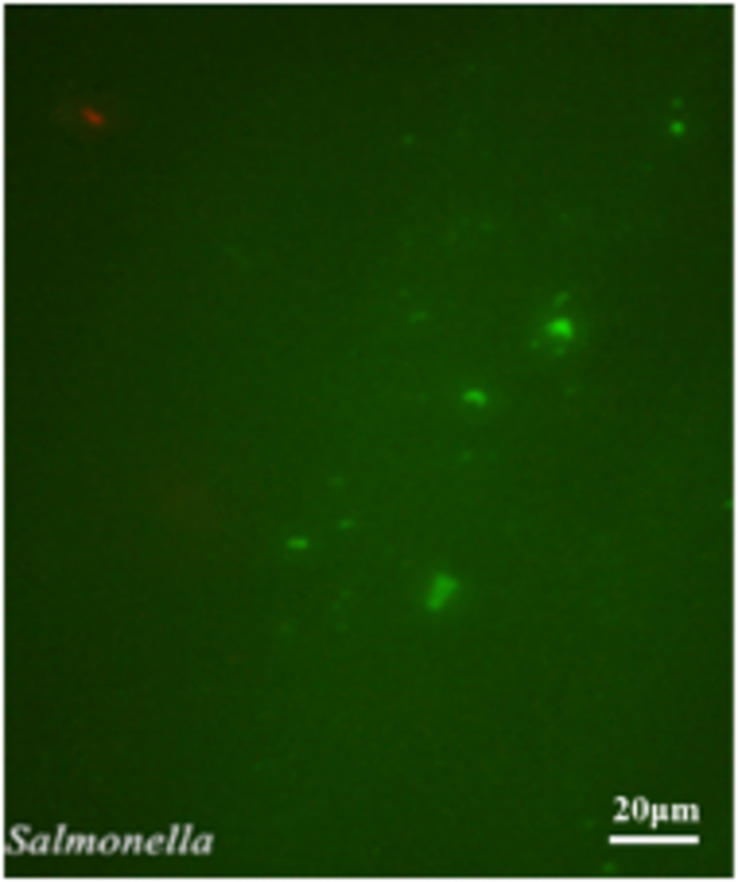
The observation of unculturable *Salmonella* cells by fluorescence microscope.

The exponential-phase *S. enterica* cells were induced to a VBNC state by low-temperature storage (Figure 1). After 30 days of storage at 4° or −20°C, the culturable number dropped to 0. As fluorescent green cells could be captured by microscopy, *S. enterica* was considered to be successfully induced into a VBNC status (Figure 2), with live and dead cells that coexisted.

### Effects of Environmental Conditions on *S. enterica* VBNC State

#### Culturable Number of *S. enterica* Cells

**FIGURE 3:**
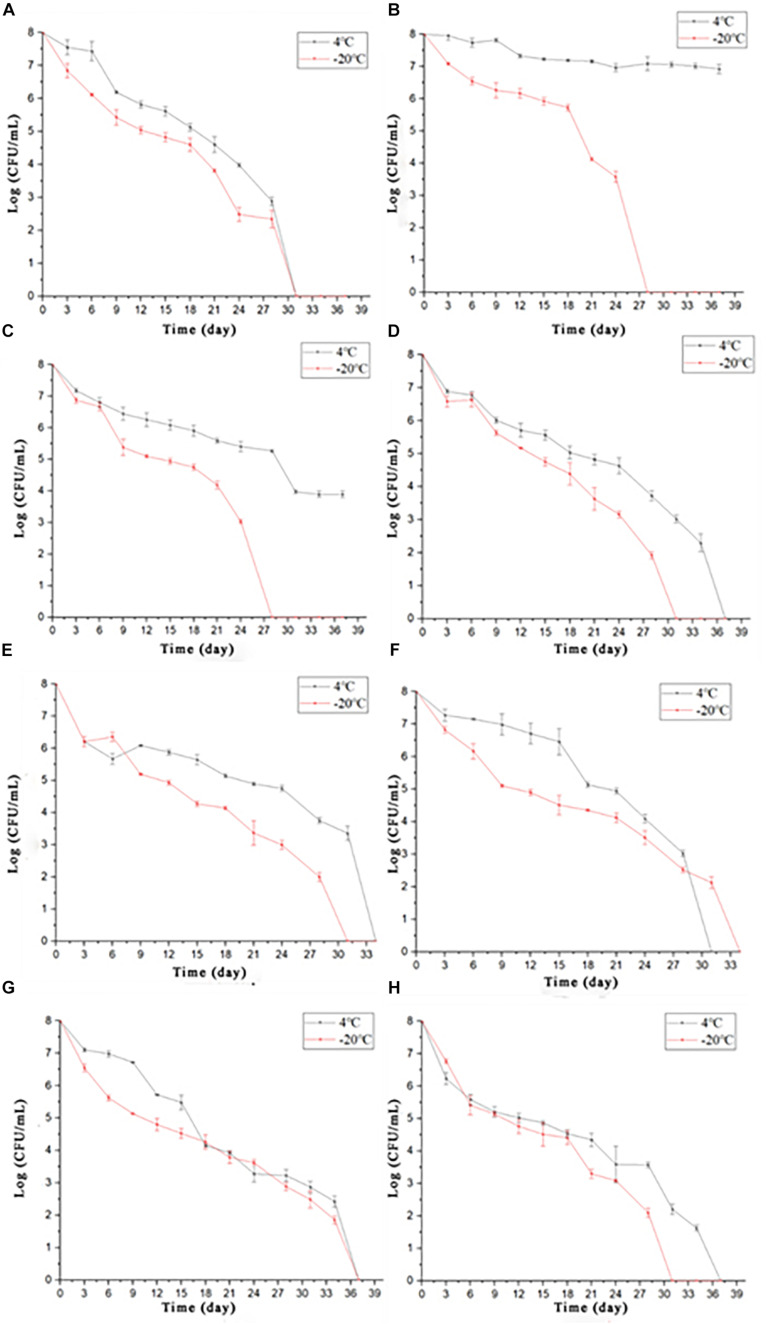
The culturable number of *Salmonella* stored under 16 different conditions (**A–H** were the culturable number tendency of *Salmonella* under correspondent conditions according to methods 1, 2, 5, 7, 9, 10, 13, and 14 and stored at 4° or −20°C, respectively).

According to four external environmental factors (nutrient, salinity, acidity, and temperature), the orthogonal array was designed to induce *S. enterica* VBNC cells including 16 groups (Table 4). Non-culturable cells were found in eight groups (groups 3, 4, 6, 8, 11, 12, 15, and 16) after 3 days of induction. Seven groups (groups 3, 4, 8, 11, 12, 15, and 16) had a high concentration (≥0.7% v/v) of acetate, indicating that the viability of *S. enterica* might be mainly inhibited by high acidity. When supplied with adequate nutrition, the *S. enterica* cells could survive under acidic stress from 0.7% v/v acetate within 30 days (group 7), suggesting that nutrition may be a stimulated bacterial stress response mechanism to resist acetate. The culturable number in the other groups (groups 1, 7, 9, 10, 13, and 14) showed a decreasing trend to reach 0 (Figure 3). The accurate time required for each protocol to reach a non-culturable cell state at 4°C and −20°C differed (Table 5). The cells grown in different concentrations of nutrients (groups 2, 5, 7, 9, 10, 13, and 14) could survive for more than 30 days. However, the result of group 4 showed that under a nutrient-rich condition (100%), high acidic stress (1% v/v acetate) still inactivated *S. enterica* cells within 3 days of storage, revealing that the effect of acidity on cell viability was stronger than nutrients. Interestingly, in protocols 2 and 5, the trend of culturable cell numbers had a significant difference, as the *S. enterica* remained at cell numbers higher than 10^4^ CFU/ml at 4°C rather than at −20°C. This phenomenon demonstrated that freezing conditions (−20°C) might contribute to inhibit bacterial growth.

**TABLE 4:** The experimental methods of orthogonal array design of VBNC induction of *Salmonella.*

**Group**	**TSB (%)**	**NaCl (%)**	**Acetate (%)**
1	0	0.9	0
2	25	0.9	0.3
3	50	0.9	0.7
4	100	0.9	1
5	25	10	0
6	0	10	0.3
7	100	10	0.7
8	50	10	1
9	50	20	0
10	100	20	0.3
11	0	20	0.7
12	25	20	1
13	100	30	0
14	50	30	0.3
15	25	30	0.7
16	0	30	1

**TABLE 5:** The time of culturable number of *Salmonella* decreased to 0 stored at different protocols.

**Group**	**4°C**	**−20°C**	**Group**	**4°C**	**−20°C**
1	31 d	31 d	9	34 d	31 d
2	+	28 d	10	31 d	34 d
3	/	/	11	/	/
4	/	/	12	/	/
5	+	28 d	13	37 d	37 d
6	/	/	14	37 d	31 d
7	37 d	31 d	15	/	/
8	/	/	16	/	/

*“+” represents cultivable; “/” represents non-culturable within 3 days.*

In summary, under higher than 0.7% v/v of acetate, the order of environmental conditions in affecting the survival of *S. enterica* was as follows: acidity > nutrients > salt. With the reduction in acidity, the supplement of certain nutrients promoted *S. enterica* to resist environmental stress.

#### *Salmonella enterica* VBNC State Formation

**FIGURE 4:**
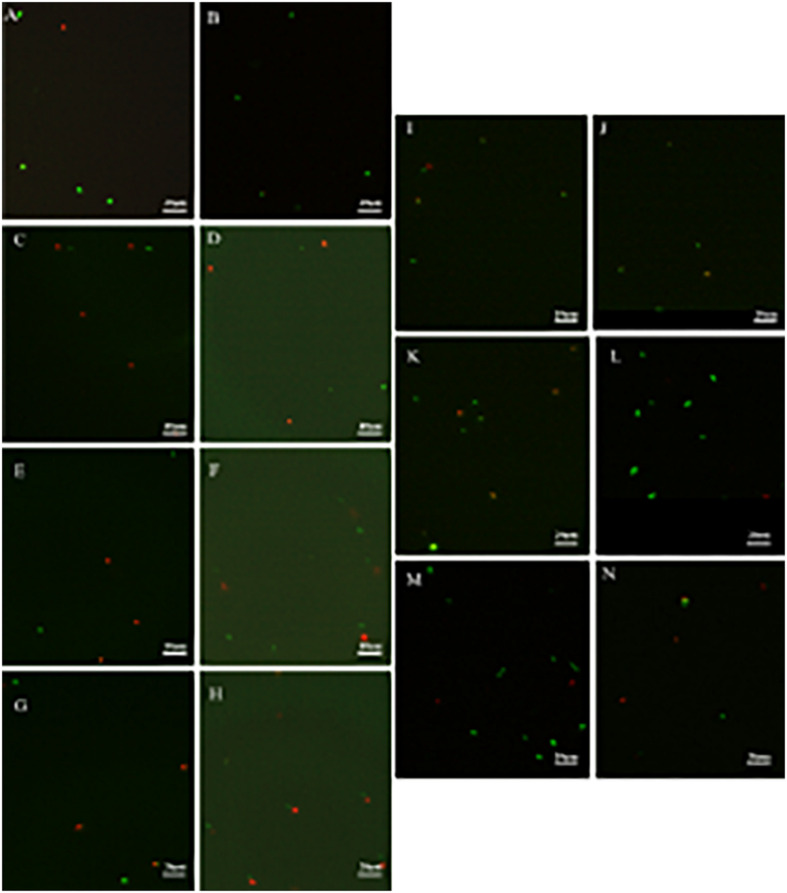
The viability of non-culturable *Salmonella* stored at different conditions by fluorescent observation (**A,B**: group 1; **C:** group 2; **D:** group 5; **E,F**: group 7; **G,H**: group 9; **I,J**: group 10; **K,L**: group 13; **M,N**: group 14).

Although *S. enterica* were no longer culturable in eight groups (groups 1, 2, 5, 7, 9, 10, 13, and 14), the viable cells were still captured by fluorescence microscopy (Figure 4). These results demonstrated that VBNC cells were successfully induced by freezing conditions (−20°C) with long-term storage (∼30 days). As none of viable *S. enterica* cells were observed from other groups (groups 3, 4, 6, 8, 11, 12, 15, and 16) in which bacteria lost culturability within a short-term storage (∼3 days) (data not shown), the formation of a VBNC state might require *S. enterica* to suffer by long-term induction of sublethal environmental stress and low temperature.

Most non-viable cell groups contained more than 0.7% v/v acetate, indicating that high-level acidity environmental condition induced cell death rather than the formation of a VBNC state, although a previous study had reported that an *S. enterica* VBNC state could be induced by lactic acid or peracetic acid (Purevdorj-Gage et al., 2018). Interestingly, a VBNC state was induced under 0.7% v/v acetate by supplying sufficient nutrients (100%), suggesting that nutrients were essential for *S. enterica* entering a VBNC state in response to multistress conditions including inorganic salts and weak acid.

As described by Chen et al. (2019) more than 10 or 1% of *S. enterica* cells were induced into a VBNC state without nutrients at 4° or −20°C, respectively. In our study, *S. enterica* entered into a VBNC state under starvation condition at 4° or −20°C (groups 1, 2, 5, 7, 9, 10, 13, and 14). Other previous studies had described similar results and showed that temperature upshift and growth factors (catalase and Tween 20) were required for resuscitation (Gupte et al., 2003; Zeng et al., 2013). Moreover, groups 2 and 5 showed that a decreasing temperature could accelerate the reduction of culturable cells and the generation of VBNC cells, which revealed that the decrease in temperature was one of the essential inducers. Besides, consistent with previous report, our results showed that the generation of *S. enterica* VBNC cells could be induced by salt stress with different concentrations, indicating that salt stress was another inducer for a VBNC state formation (Asakura et al., 2002).

Together, four inducers including nutrition starvation, salt stress, low-level acidity, and low temperature were concluded for VBNC state induction. However, during induction with multistress conditions, nutrition starvation antagonizes with low-level acidity. Besides, high-level acidity was considered as an inhibitor for VBNC induction. Therefore, the keynote environmental factors of VBNC state induction were concluded to be: (i) nutrient-rich acidic environment, (ii) oligotrophic low-acidity environment, and (iii) oligotrophic refrigerated environment.

### Effects of Keynote Environmental Factors on Salmonella VBNC State Formation

#### Acidity

Acetate had been utilized as an antimicrobial chemical for many years in food production. As previously described by Liao et al. (2003) exposing *Salmonella* cells to 0.7 or 1.0% acetic acid for 7 min usually caused 90% cells with inactivation and 99% of culturable cells with injury. In agreement with a related study, cell viability was evaluated based on cultivability, neglecting the generation of *Salmonella* VBNC cells induced by acetate *in vitro* (Alvarez-Ordonez et al., 2010). Encountering organic acid stress causing the acidification of the cytoplasm and the accumulation of intracellular anion, the available energy is required for *S. enterica* to efflux protons (H^+^) by active transport maintaining intracellular pH homeostasis (Mani-López et al., 2012). Therefore, the effect of acidity (0.7 or 1.0% v/v) on the formation of an *S. enterica* VBNC state in flour food was investigated with different nutritional concentrations at low temperature (4° or −20°C).

Under oligotrophic conditions, *S. enterica* could not be cultured after 3 days of storage at low temperature (4° and −20°C) by adding 0.7 or 1.0% v/v acetic acid (Table 6). The bacterial activity test results showed that only dead cells were observed, indicating that *S. enterica* was unable to enter into a VBNC state. When supplied with sufficient nutrients (≥50%), the culturable cells still existed under 0.7% v/v acetic acid after 3 days of storage, indicating that acid tolerance of *S. enterica* was improved by available nutrients, which might ultimately lead to generation of VBNC cells. However, when the concentration of acetate reached 1.0% v/v, no culturable cells could be found after 3 days of storage, whether provided with nutrients or not. As expected, the activity test results showed that all the bacterial cells were dead, indicating that the formation of a VBNC state of *S. enterica* could be controlled by adding 1.0% v/v acetate, even when supplied with rich nutrients. Interestingly, only group 7 had showed that all the bacteria were inviable with decreasing temperature under high-level acidity stress (1.0% v/v acetate). These results were consistent with those previously described by Alvarez-Ordonez et al. (2010), who measured the organic acid tolerance of *S. typhimurium* at different growth temperatures and found that the reduction of low temperatures markedly decreased the acid resistance and increased the growth pH boundary of *S. typhimurium*. In summary, during the processing of flour foods, 1.0% v/v acetic acid could be used to clean the processing equipment, which can effectively eliminate the pollution of *S. enterica* and its VBNC cells.

**TABLE 6:** Inhibition of acidity in the formation of VBNC state of *Salmonella.*

**Group**	**TSB (%)**	**NaCl (%)**	**Acetate (%)**	**Cultivability**		**Viability**	
				**4°C**	**−20°C**	**4°C**	**−20°C**
1	0	0.9	0.7	/	/	–	–
2			1.0	/	/	–	–
3	25	0.9	0.7	/	/	–	–
4			1.0	/	/	–	–
5	25	10	0.7	/	/	–	–
6			1.0	/	/	–	–
7	100	10	1.0	+	/	ND	–
8	50	20	0.7	+	+	ND	ND
9			1.0	/	/	–	–
10	100	20	0.7	+	+	ND	ND
11			1.0	/	/	–	–
12	100	30	0.7	+	+	ND	ND
13			1.0	/	/	–	–
14	50	30	0.7	+	+	ND	ND
15			1.0	/	/	–	–

*“+” represents culturable; “/” represents non-culturable; “–” represents inactivation; “ND” represents non-detection.*

#### Nutrients

Recently, Pu et al. (2019) proposed that protein aggresome is an important indicator of the *E. coli* VBNC state, which was promoted by nutrient starvation, but stress removal will facilitate the disaggregation of the proteins by the DnaK–ClpB cochaperone system (Pu et al., 2019). Although nutrients also play an important role in the formation of the *S. enterica* VBNC state, the reduction of nutrients may be a potential method to inhibit *S. enterica* VBNC cell formation. Under high salt and low acidity, *S. enterica* cells were still culturable with an oligotrophic condition after 3 days of storage (Table 7), which suggested that only an oligotrophic condition was incapable of inhibiting the formation of VBNC cells. Therefore, it is essential to combine oligotrophic condition with other environmental stress (e.g., high acidity) to prevent *S. enterica* from entering the state of VBNC.

**TABLE 7:** Inhibition of nutritional status in the formation of VBNC state of *Salmonella.*

**Group**	**TSB (%)**	**NaCl (%)**	**Acetate (%)**	**Cultivability**		**Viability**	
				**4°C**	**−20°C**	**4°C**	**−20°C**
1	0	20	0	+	+	ND	ND
2	25			+	+	ND	ND
3	0	20	0.3	+	+	ND	ND
4	25			+	+	ND	ND
5	0	30	0	+	+	ND	ND
6	25			+	+	ND	ND
7	0	30	0.3	+	+	ND	ND
8	25			+	+	ND	ND

*“+” represents culturable; “ND” represents non-detection.*

### Control and Reduction of *S. enterica* VBNC Cells in Flour Food

**FIGURE 5:**
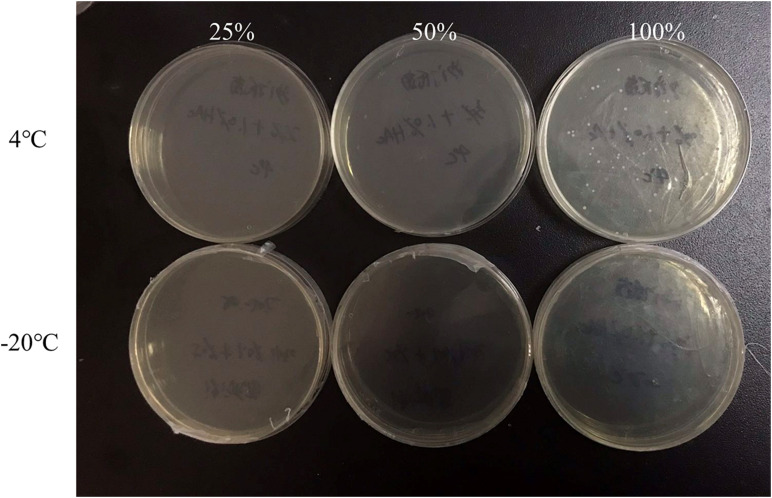
The culturable number of *Salmonella* inoculated in the 1.0% (v/v) acetate medium containing 100, 50, and 25% nutrients at low temperature for 3 days.

**FIGURE 6:**
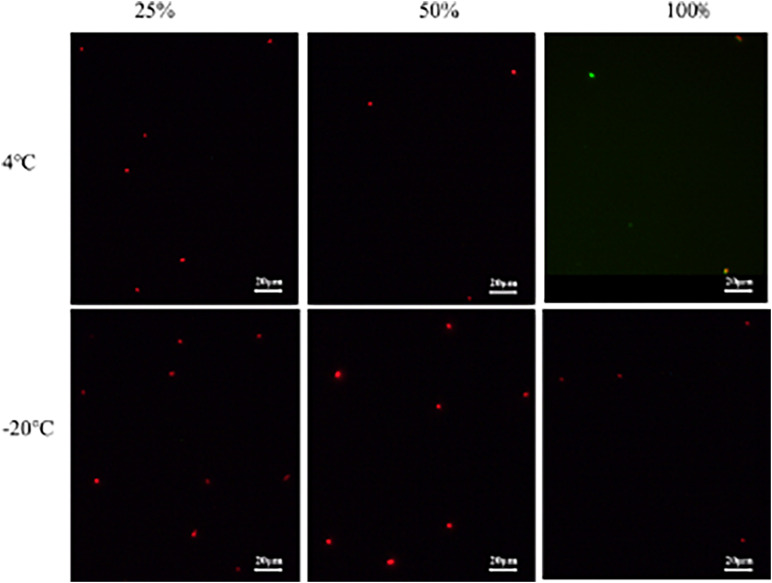
The viability of unculturable *Salmonella* stored at different conditions with fluorescent observation.

In order to confirm the inhibitory effect of the above keynote conditions on *Salmonella* VBNC formation in food samples, we selected crystal cake powders as a sole source of nutrients instead of TSB to simulate the flour food environment. The keynote conditions used for investigation was high acidity (1.0% v/v acetate) combined with different concentrations (25, 50, and 100%) of nutrients. After 3 days of storage, the culturable number and viability of *S. enterica* in different conditions are shown in Figures 5, 6. Consistent with the results from non-food systems, only dead bacterial cells could be found in simulated food systems after 3 days of storage at −20°C, whether they were provided with nutrients or not (Figure 6). On the contrary, *Salmonella* could still survive after 3 days of storage at 4°C via a supplement of sufficient nutrients (100%) (Figure 6). Although the number of survival of *S. enterica* cells was significantly reduced, there is a possibility of culturable cells entering the VBNC state (Figure 5). Therefore, the best control conditions for the formation of VBNC status of *S. enterica* in flour food is to add 1.0% v/v acetic acid combined with storage at −20°C.

Foodborne pathogenic and spoilage bacteria had been previously shown to enter into a VBNC state in a food system under a freezing environment. Therefore, the results of this study might provide a theoretical basis for the control and reduction of foodborne bacterial VBNC cells.

### PMA–PCR Detection on VBNC Cells

In order to eliminate the interference of dead bacterial DNA, samples were subjected to PMA treatment before PCR amplification. The detection limit of *S. enterica* using the constructed PMA–PCR technology to detect the VBNC status in the crystal cake food system is 10^5^ CFU/ml, which is consistent with the PMA–PCR results in the pure induction system (data not shown). Compared to the detection limit of PCR for culturable *S. enterica* in food systems, the detection limit of PMA–PCR has no significant changes, suggesting that the PCR method by the PMA dye is non-effective. Therefore, the PMA–PCR method can be better applied to detect viable bacteria (culturable and non-culturable) in a food sample, preventing false-negative detection of a culture-based method on VBNC cells.

## Conclusion

In this study, the influence factors including nutrition, acid, salt, and temperature for the entry into a VBNC state of *S. enterica* and an efficient detection method were investigated. The order of environmental conditions in effecting the cultivability of *S. enterica* was as follows: acidity > nutrients > salt. Four inducers for the VBNC state including nutrition starvation, salt stress, low-level acidity, and low temperature were concluded. However, during induction with multistress conditions, nutrition starvation antagonizes with low-level acidity. Besides, high-level acidity was considered as an inhibitor for a VBNC state formation. Therefore, the keynote conditions for *S. enterica* entering the VBNC state were concluded as (i) nutrient-rich acidic environment, (ii) oligotrophic low-acidity environment, and (iii) oligotrophic refrigerated environment. Thus, using an environment condition of high acidity (1.0% v/v acetate) with low temperature (−20°C), the formation of *S. enterica* VBNC state was eliminated in flour food. Combined with PMA pretreatment, the PCR technology could be applied to detect viable *S. enterica* cells (culturable and VBNC) removing the interference of dead cells. The detection limit of the PMA–PCR technology was 10^5^ CFU/ml in an artificially simulated food system. In conclusion, this study identified specific environmental stresses to control, and applied a stable PMA–PCR method to detect, an *S. enterica* VBNC state, providing a theoretical basis for the control and reduction of foodborne bacterial VBNC cells.

## Data Availability Statement

All datasets presented in this study are included in the article/supplementary material.

## Author Contributions

JL and KW conceived of the study and participated in its design and coordination. JF and TH performed the experimental work. CB and LC analyzed the data. YL and JL prepared and revised this manuscript. All authors reviewed and approved the final manuscript.

## Conflict of Interest

YL was employed by the company Guangdong Zhongqing Font Biochemical Science and Technology Co. Ltd.

The remaining authors declare that the research was conducted in the absence of any commercial or financial relationships that could be construed as a potential conflict of interest.
